# Gut microbiota-derived trimethylamine N-oxide is associated with the risk of all-cause and cardiovascular mortality in patients with chronic kidney disease: a systematic review and dose-response meta-analysis

**DOI:** 10.1080/07853890.2023.2215542

**Published:** 2023-05-29

**Authors:** Yachun Li, Hongmei Lu, Jing Guo, Meiling Zhang, Huijuan Zheng, Yuning Liu, Weijing Liu

**Affiliations:** a Dongzhimen Hospital, Beijing University of Chinese Medicine, Beijing University of Chinese Medicine, Beijing, P.R. China; b Key Laboratory of Chinese Internal Medicine of Ministry of Education and Beijing, Beijing, P.R. China; c Zhanjiang Key Laboratory of Prevention and Management of Chronic Kidney Disease, Guangdong Medical University, Zhanjiang, Guangdong, P.R. China

**Keywords:** Trimethylamine N-oxide, chronic kidney disease, all-cause mortality, cardiovascular mortality

## Abstract

**Background:**

Trimethylamine N-oxide (TMAO) derived from gut microbiota causes kidney-heart damage in chronic kidney disease (CKD) patients. However, it is controversial whether CKD patients with higher TMAO are associated with a higher risk of death. We aimed to assess the correlation between circulating TMAO concentration and the risk of all-cause and cardiovascular death in CKD patients of different dialysis statuses and different races by dose-response analyses, and the underlying mechanisms were also explored by analyzing the correlations of TMAO with glomerular filtration rate (GFR) and inflammation.

**Method:**

PubMed, Web of Science, and EMBASE were systematically searched up to 1 July 2022. A total of 21 studies involving 15,637 individuals were included. Stata 15.0 was used to perform the meta-analyses and dose-response analyses with extracted data. Subgroup analyses were conducted to recognize possible sources of heterogeneity.

**Results:**

The risk of all-cause mortality was increased in non-dialysis CKD patients (RR = 1.26, 95%CI = 1.03–1.54, *p* = 0.028) and non-black dialysis patients (RR = 1.62, 95%CI = 1.19–2.22, *p* = 0.002) with the highest circulating TMAO concentration, and the association was confirmed to be linear. In addition, an increased risk of cardiovascular mortality was also found in non-black dialysis patients with the highest circulating TMAO concentration (RR = 1.72, 95%CI = 1.19–2.47, *p* = 0.004), likewise, a linear association was identified. However, for dialysis patients including blacks with high TMAO concentrations, there was no significant increase in either all-cause mortality (RR = 0.98, 95%CI = 0.94-1.03, *p* = 0.542) or cardiovascular mortality (RR = 0.87, 95% CI = 0.65–1.17, *p* = 0.362). Meanwhile, we verified strong correlations between TMAO and both GFR (*r*= −0.49; 95% CI= −0.75, −0.24; *p* < 0.001) and inflammatory markers (*r* = 0.43; 95% CI= 0.03, 0.84; *p* = 0.036) in non-dialysis patients.

**Conclusions:**

Increased circulating TMAO concentrations increase the risk of all-cause mortality in non-dialysis and non-black dialysis CKD patients. Moreover, elevated TMAO levels raise the cardiovascular mortality risk in non-black dialysis patients.

**Key messages**
1. Non-dialysis and non-black dialysis CKD patients with higher circulating TMAO concentrations are associated with an increased risk of all-cause mortality.
2. Non-black dialysis patients with higher concentrations of TMAO are associated with an increased risk of cardiovascular mortality.
3. Circulating TMAO concentrations have a strong negative correlation with GFR and a positive correlation with inflammation biomarkers in non-dialysis CKD patients.

## Introduction

Chronic kidney disease (CKD) is a major threat to human health, which can cause multiple organs and system complications, and eventually develop into end-stage renal disease, which requires continuous dialysis or kidney transplantation, resulting in a decline in the quality of life and a heavy socioeconomic burden [1]. In 2017, there were about 697.5 million CKD patients nationwide, with a prevalence rate of 9.1%, of which 1.2 million people died from CKD. Compared with 1990, the all-age mortality rate from CKD increased by 41.5% [2]. Furthermore, cardiovascular mortality is significantly higher in individuals with CKD. [3] Therefore, the prevention and treatment of CKD is becoming more and more urgent.

The gut-kidney-heart axis in chronic kidney disease has gradually attracted extensive attention [4,5], that is, the accumulation of gut microbial-derived uremic toxins leads to the occurrence and development of CKD, and increases the risk of cardiovascular events in CKD. Trimethylamine-N-oxide (TMAO), derived from dietary intake of animal-derived choline, L-carnitine, and plant-derived betaine, is one of the uremic toxins that cause kidney-heart damage. When food is transported to the intestinal lumen, it is metabolized by the intestinal flora to produce trimethylamine (TMA), which is absorbed through the hepatic portal venous system and converted to TMAO by flavin-containing monooxygenase (FMO) [6,7]. As we all know, the number of microbes that colonize the human gastrointestinal tract is 10 times greater than the number of the host’s own somatic and germ cells [8], which defends against harmful substances and also produces absorbable nutrients. However, the gut flora in CKD patients becomes unbalanced and disordered [9–12]. It is confirmed that the abundance of TMA-producing gut microbiota is increased in patients with T2DM-CKD [13]. Under physiological conditions, circulating TMAO is excreted almost exclusively by the kidneys in the form of glomerular filtration and tubular secretion [7,14]. Thus, disruption of kidney function, together with dysbiosis of gut microbiota, leads to increased circulating TMAO concentrations in patients with CKD including end-stage kidney disease (ESKD) [15–17], which in turn exacerbates kidney damage by promoting inflammation [18–21].

Based on the pro-inflammatory and pro-fibrotic effects of TMAO, the association between TMAO and mortality in CKD patients has been a well-concerning focus over recent years, while the findings are not completely uniform. Studies have shown that TMAO level is an independent risk factor for death (5-year survival rate) in patients with CKD 3-5 [22] and for hospitalization events in patients receiving maintenance hemodialysis [23]. On the contrary, no significant correlation was found between plasma TMAO concentrations and the progression of disease or mortality in patients with CKD after adjusting for confounders [24]. Therefore, we performed the current systematic review and meta-analyses to reveal the dose-response association between circulating TMAO concentrations and mortality. Meanwhile, from the perspective of etiology, the correlations of TMAO with renal function and inflammatory biomarkers in CKD patients were studied. To our knowledge, this is the first report to study dialysis and non-dialysis patients separately, which greatly increases the specificity of results.

## Methods

The protocol of the recent study has been registered in the International prospective register of systematic reviews (PROSPERO) with the identification number CRD42022321968.

### Data source and search strategy

A systematic search was performed in Web of Science, PubMed, and EMBASE through 1 July 2022. The literature search was carried out by the combination of Medical Subject Headings (MeSH) and free-text terms, with no date and language restrictions. Search strategies are presented in Supplementary Table 1. The reference list of all relevant articles and reviews is also manually searched to ensure that all relevant literature was included. Two authors searched the literature (YC. Li and HM. Lu), and two other authors (J. Guo and ML. Zhang) reviewed uncertain cases. The final decisions were made by consensus.

### Study selection

The relevant observational studies with cohort, case-control, nested case-control, or analytic cross-sectional studies were included in the current review if they: (1) included CKD patients; (2) involved participants aged >18 y; (3) reported circulating TMAO concentrations for at least 2 and 3 categories and conducted 2-class and dose-response meta-analysis, respectively; (4) reported the correlation between circulating TMAO concentrations and GFR; (5) reported the correlation between circulating TMAO concentrations and inflammation-related indicators. Interventional studies, animal studies, and studies involving pregnant women and other serious diseases such as cancer or immune-related diseases were excluded.

### Data extraction and quality assessment

The quality of all studies was assessed independently by two authors (YC. Li and HM. Lu). For studies with controversial evaluation results, we consulted with the third investigator (HJ. Zheng) to make the decision. The Newcastle–Ottawa Scale (NOS) [25] and the Agency for Healthcare Research and Quality (AHRQ) [26] checklist was used to assess the quality of cohort studies and cross-sectional studies, respectively. The items were scored ‘1’ if the answer was ‘YES’, and ‘0’ if the answer was ‘NO’ or ‘UNCLEAR’. The final quality assessment scores were as follows: low quality=0–3; moderate quality=4–7; high quality=8–11. In addition, the Quality in Prognosis Studies (QUIPS) tool, which is considered to be the newest and most robust method of assessing the risk of bias in systematic reviews/meta-analyses, was also used to evaluate the inexpensive risks of prognostic studies.

The standard extraction form was used to extract the data by two investigators (J. Guo and ML. Zhang) to independently obtain the below information: study characteristics (first author, publication year, location of the study, sample size, follow-up time, and study design), baseline characteristics of participants (age, gender, and disease status), TMAO characteristics (sample source from serum or plasma, and median, mean or range in each category of TMAO concentrations), and adjusted confounders. In addition, the number of deaths, sample size as well as HR, and 95% CI in each category of TMAO were extracted to conduct dose-response analyses. If relevant data were not directly reported in the original literature, the number of deaths and HR were estimated from survival curves. Besides, the *r* or *rho* values between TMAO concentration and GRF or inflammatory biomarkers were also recorded for meta-analyses of the correlations.

### 2.4. Data synthesis and analysis

First, we performed meta-analyses of the HRs of all-cause mortality and cardiovascular mortality in the category of highest TMAO concentration category. Second, dose-response analyses require the median or mean of TMAO concentrations for each category, which was preferentially extracted. If not given directly, data were calculated as the median of the TMAO concentration range for each category. If the TMAO concentration is an open interval, it is derived using the interval of adjacent classes. Third, for correlation analyses, confidence intervals are calculated after converting the correlation coefficient (*r*) to fisher’*Z*, and then converting back to the correlation coefficient (*r*) [27].

Furthermore, we performed a heterogeneity test for each effect size, and significant statistical heterogeneity was affirmed if *I*^2^>50%. Riley et al. [28] proposed that the random-effects model was considered the best method to assess the effect size, because of the inconsistencies in study design, sample source, or the number of patients in all included studies. In this case, a random effect model was used for meta-analyses. A variety of different subgroup analyses were conducted to explore sources of literature heterogeneity. Funnel plots were used to assess the publication bias. If the number of articles included to assess each effect size is less than 10, the detection efficiency of publication bias is too low to be tested [29].

## Results

### Literature search and study characteristics

**Figure 1.**
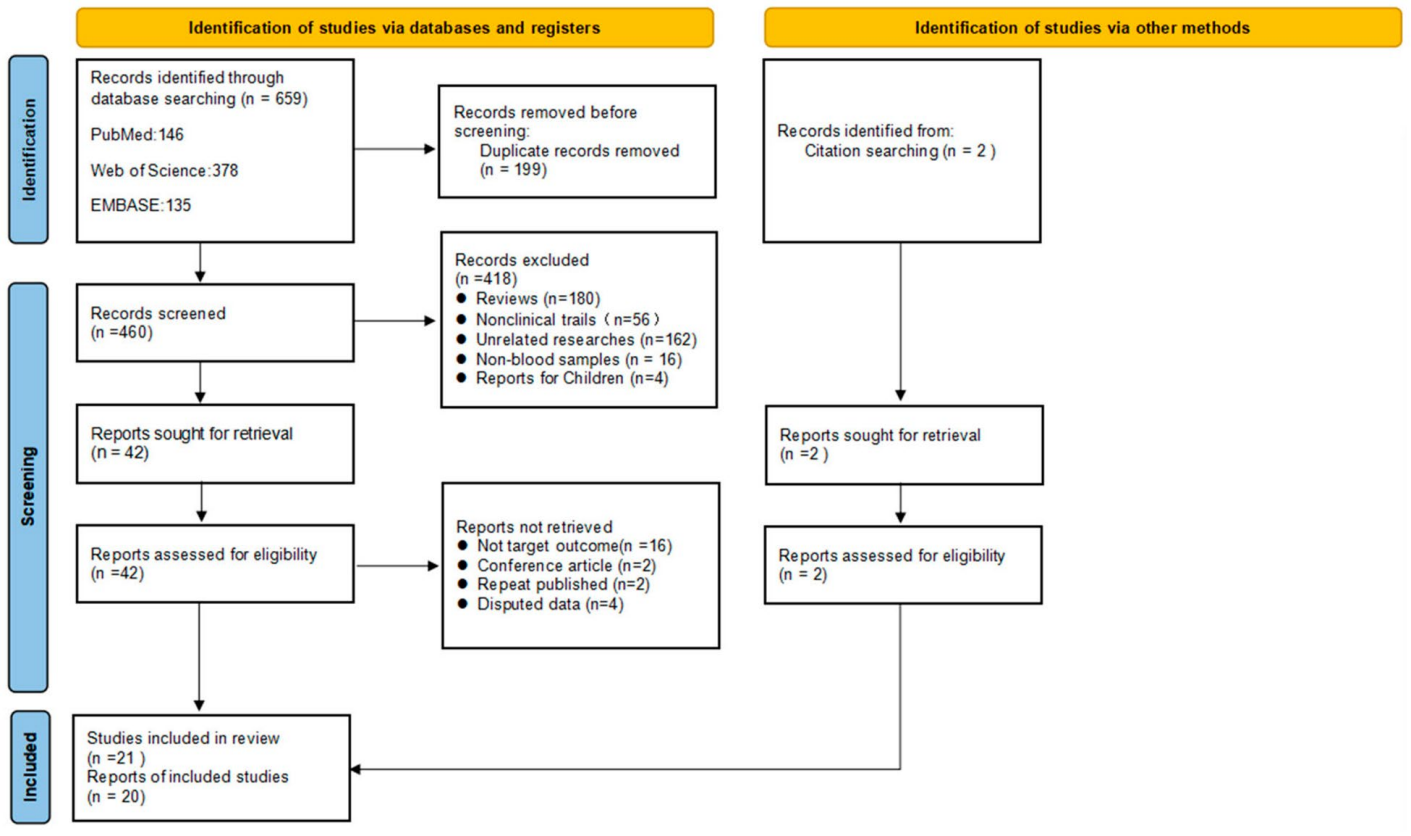
Flow chart of the literature search and study selection process.

A total of 691 articles were retrieved, and 462 articles remained after removing duplicate articles, which were screened by reading the title and abstract. 50 literature were left for full-text assessment. Finally, we included 21 studies for meta-analyses. According to the consistency test of the results of the two authors on whether the literature was included or not, the kappa value was 0.836, indicating a high consistency of the included literature. The flow chart of literature search and screening has been presented in Figure 1.

The characteristics of the included studies and the demographic and mean baseline parameters of the patients are summarized in Table 1. We included studies from almost all over the world, with 5 from China and Japan in Asia [33,47,41,42,44], 9 from the US and Canada in North America [24,30,35,31,32,34,13,36,37,46], 5 from Sweden, the Netherlands and Denmark in Europe [22,34,39,43,45], and 1 from Egypt in Africa [40]. The research type was mainly cohort with 17 studies, and cross-sectional with 3 studies [33,13,40]. The average age of the participants ranged from 46 to 70 years old. Median follow-up time fluctuated in the range of 2.3–15 years. All studies included both males and females.

**Table 1.** Characteristics of the selected studies.

First author	Year/ Country	Study design	Subjects (% male)	Age (years)	Follow-up (years)	Disease status	Sample Source	Events for analysis	Adjusted confounders
Tang [30]	2015/ United States	Cohort	521 (48.0)	70 (10)	5	Non-dialysis CKD	Plasma	All-cause mortality, GFR, inflammatory biomarkers	Age, gender, SBP, low-density lipoprotein cholesterol, high-density lipoprotein cholesterol, smoking, DM, hsCRP, and eGFR
Kaysen [31]	2015/ United States	Cohort	235 (55.3)	61.8 (14.2)	4	HD	Serum	All-cause mortality, inflammatory biomarkers	_
Stubbs [32]	2016/ United States	Cross-sectional	56 (50)	<60	—	Non-dialysis CKD and HD	Serum	GFR	_
Missailidis [22]	2016/ Sweden	Cohort	170 9(65)	55 (14)	5	Non-dialysis CKD	Serum	All-cause mortality, GFR, inflammatory biomarkers	Gender, age,DM,hsCRP,GFR
Robinson- Cohen [24]	2016/ United States	Cohort	339 (68.4)	57.3 (13.5)	3.3	Non-dialysis CKD	Plasma	All-cause mortality	Age, race, sex, SBP, LDL, HDL, Smoking, CRP, eGFR
Mafune [33]	2016/Japan	Cross-sectional	227(70)	68 (61–74)	_	Non-dialysis CKD	Serum	GFR	_
Kim [34]	2016/Canada	Cohort	2529 (62.5)	68.2 (12.7)	3	Non-dialysis CKD	Plasma	GFR	_
Al-Obaide [13]	2017/ United States	Cross-sectional	20(/)	64.4(2.3)	_	Non-dialysis T2DM and CKD	Serum	Inflammatory biomarkers	_
Gruppen [35]	2017/ Netherlands	Cohort	5469 (48.7)	<60	8.3	Non-dialysis CKD	Plasm	All-cause mortality, GFR	Age, sex, body mass index, smoking, type 2 DM, history of CVD, history of cancer, anti-hypertensive medication, lipid-lowering drugs, SBP, total cholesterol, high density lipoprotein cholesterol and triglycerides, UAE, eGFR crea-cysC
Shafi [36]	2017/ United States	Cohort	1232 (43.3)	57.7 (13.8)	2.3	HD	Serum	All-cause mortality, cardiovascular mortality	Age, sex, ICED severity score, cause of ESRD, BMI, SBP, albumin, and relative calculated from urinary urea clearance
Stubbs [37]	2019/ United States	Cohort	1243 [38]	54 (14)	5.3	HD	Serum	All-cause mortality, cardiovascular mortality	Age, sex, BMI, SBP, albumin, race, dialysis duration, history of smoking, history of cardiovascular disease, history of percutaneous coronary intervention, history of stroke, history of MI, BUN, and the Evaluation of Cinacalcet Hydrochloride Therapy to Lower Cardiovascular Events study design
Winther [39]	2019/ Denmark	Cohort	484 (-)	46(13)	15	Type 1-diabetes and macroalbuminuria	Plasma	All-cause mortality, cardiovascular mortality	Age, sex, diabetes duration, HbA_1c_, SBP, total cholesterol, smoking status, and UAER
EI-Deeb [40]	2019/Egypt	Cross-sectional	80 (48.8)	<60	_	Non-dialysis CKD	Plasma	GFR, inflammatory biomarkers	_
Zhang [41]	2020/China	Cohort	252 (56)	57.1 (14.5)	6.1	HD	Plasma	All-cause mortality, cardiovascular mortality	Age, sex, DM, BMI and rGFR
Fu [42]	2021/China	Cohort	1032(57)	48 (14)	5.3	PD	Serum	All-cause mortality, cardiovascular mortality	Age, sex, DM, history of CVD, BMI, MAP, albumin, TG, LDL-C, hs-CRP, rGFR, and total Kt/V
Winther [43]	2021/ Denmark	Cohort	311 (75.2)	57.2 (8.2)	6.8	type 2-diabetes and albuminuria	Plasma	All-cause mortality, cardiovascular mortality	Age, sex, HbA1c, SBP, BMI, total cholesterol, smoking, UAER, and eGFR
Chang [44]	2021/China	Cohort	513 (58.1)	54.0 (15.5)	2.9	PD	Serum	All-cause mortality, cardiovascular mortality	Age, sex, DM, CVD, BMI, Alb, hs-CRP, potassium, phosphorus, RRF, nPNA, and calendar year of the catheter implantation
Hernandez [45]	2022/ Sweden	Cohort	216 (56)	66 (51–74)	5	HD	Serum	All-cause mortality, inflammatory biomarkers	Sex, age, DM, and CVD
Sapa [46]	2022/ United States	Cohort	555 (47)	70 (9)	6.2	Non-dialysis DKD	Plasma	All-cause mortality, cardiovascular mortality, GFR	Age, sex, race, education, BMI, SBP, hypertension meds, smoking, CHD, stroke, LDL, HDL, lipid-lowering meds, UACR and eGFR
Zhang [47]	2022/China	Cohort	144 (52.8)	46 (15)	2.4	PD	Serum	Inflammatory biomarkers	Sex, age, Diabetes, BMI, and rGFR

A total of 21 studies involving 15,637 participants from 2015 to 2022 were included in our meta-analysis. After excluding 1 literature by Stubbs [32] containing both dialysis and non-dialysis patients, we included a total of 7 articles [24,30,22,35,46,39,43] to analyze the association between circulating TMAO concentrations and all-cause mortality, and 3 articles [46,39,43] to analyze the association between circulating TMAO and cardiovascular mortality, in non-dialysis CKD patients. Three out of 7 studies were conducted on patients with diabetes complicated by nephropathy, of which 1 for type 1 diabetes mellitus with proteinuria [43], 1 for type 2 diabetes mellitus with macroalbuminuria [39], and 1 for non-dialysis DKD patients [46]. In addition, 8 [31,41,42,44,36,37,45)] and 6 [41,42,44,36,37] studies only observing dialysis patients were included to analyze the association of TMAO and all-cause and cardiovascular mortality respectively. Of note, the study by Shafi et al. [36] was included as 2 independent studies due to racial differences. Of all studies on dialysis patients, 4 studies [41,42,44,36] only included non-blacks, and the remaining included black patients. Moreover, 8 [30,22,33,34,35,46,40] and 7 [30,31,22,13,47,45,40] correlation coefficients were reported between TMAO concentrations and GFR as well as inflammatory markers in non-dialysis CKD patients, respectively. Based on the results of subgrouping, we further performed dose-response dialysis of two-class variables for studies, which contains at least 3 categories of TMAO concentrations.

### Meta-analysis of the association between circulating TMAO concentrations and all-cause mortality risk

**Figure 2.**
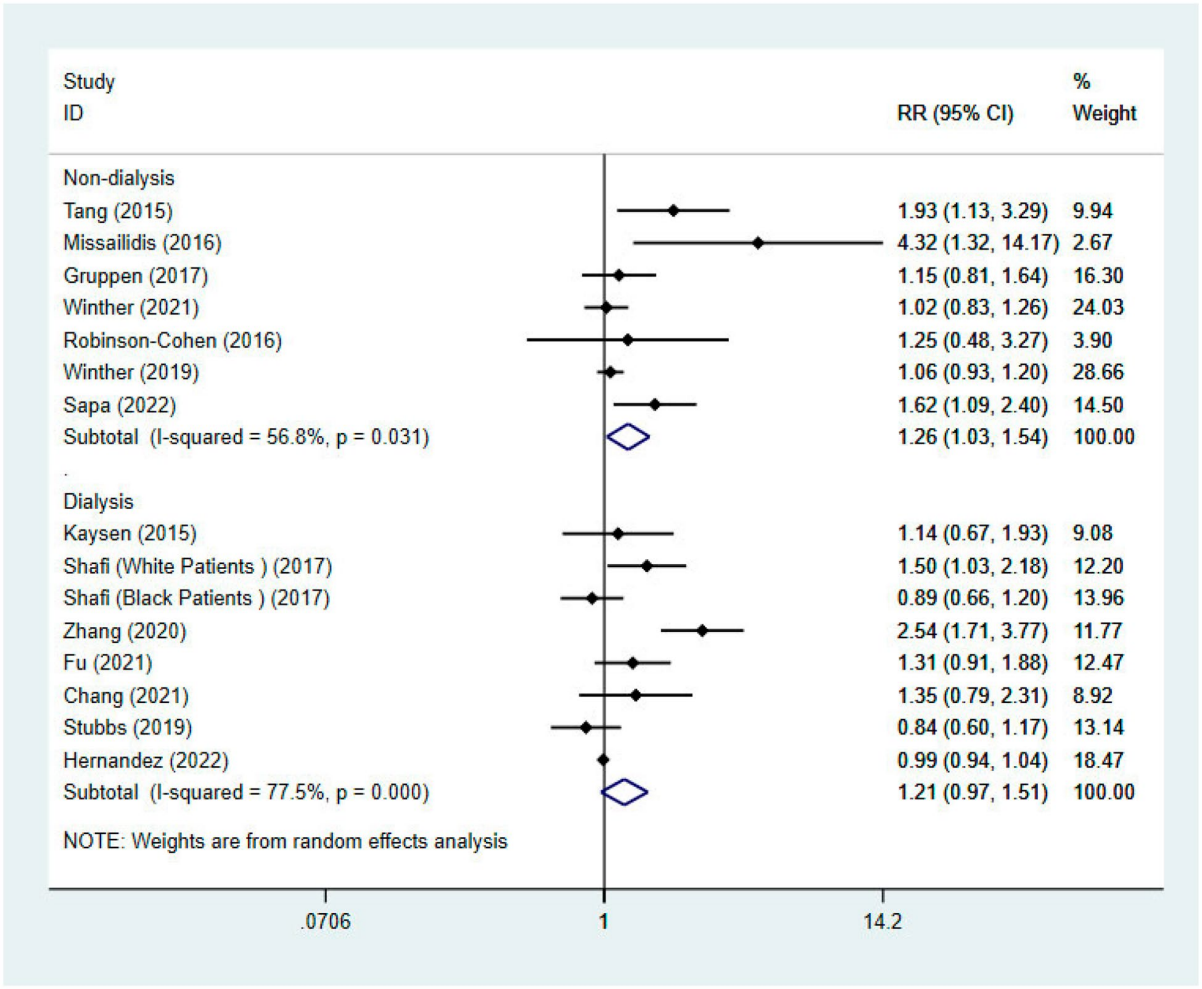
Meta-analysis of the association between circulating TMAO concentrations and all-cause mortality in non-dialysis patients and dialysis patients respectively. RR: relative risk; CI: confidence interval.

15 studies with 5628 participants were included for the analysis of the association between TMAO and all-cause mortality, 7 of which reported non-dialysis patients only [24,30,22,35,46,43]. The results showed that the risk of all-cause mortality was significantly increased in non-dialysis CKD patients with high levels of TMAO (RR = 1.26, 95%CI = 1.03–1.54, *p* = 0.028, Figure 2). Subgrouping (Table 2) according to age, GFR, and underlying diseases indeed reduced the heterogeneity, and also showed that the statistical association of circulating TMAO with the risk of all-cause mortality was not found in patients with aged <60, GFR > 60, and underlying diabetes mellitus.

**Table 2.** Subgroup analysis of the association between circulating TMAO concentrations and all-cause mortality in non-dialysis CKD patients.

Subgroup	Studies	RR	95%CI	*Z*	*P*	Heterogeneity	
						*I^2^, %*	^*^ *P*
Age							
<60	5 [26,4,43,45,40]	1.09	0.93, 1.28	1.08	0.280	30.9	0.216
>60	2 [41,39]	1.72	1.26, 2.37	3.36	0.001	0.000	0.605
GFR							
<60	4 [26,28,41,39]	1.77	1.32, 2.37	3.82	0.000	0.000	0.397
>60	3 [43,45,40]	1.06	0.95, 1.17	1.06	0.289	0.000	0.846
Underlying diseases							
CKD	4 [26,28,41,43]	1.61	1.01, 2.56	1.99	0.047	50.6	0.108
DKD	3 [39,45,40]	1.12	0.93, 1.35	1.2	0.229	54.8	0.109

*For heterogeneity among subgroups.

RR: relative risk; CI: confidence interval.

For the remaining 8 studies [31,41,42,44,36,37] on dialysis patients, we didn’t find a significant statistical association between TMAO and all-cause mortality (RR = 1.21, 95%CI = 0.97–1.51, *p* = 0.094, Figure 2). When we further performed a subgroup analysis (Table 3) by race, age, and dialysis modality, heterogeneity was significantly reduced. Interestingly, it showed that there was a notable association between the highest quartile of TMAO and the risk of all-cause mortality in 4 studies [41,42,44,36] only including non-blacks (RR = 1.62, 95%CI = 1.19–2.22, *p* = 0.002). The statistical association between TMAO and all-cause mortality was still not found in studies involving blacks (RR = 0.98, 95%CI = 0.94-1.03, *p* = 0.542). The article by Kaysen [31] mentioned that non-blacks were associated with higher mortality. This is consistent with the conclusions of our meta-analyses. In addition, subgrouping (Table 3) also revealed the association between TMAO concentrations and all-cause mortality was influenced by age of individuals, but not influenced by dialysis modality.

**Table 3.** Subgroup analysis of the association between circulating TMAO concentrations and all-cause mortality in dialysis patients.

Subgroup	Studies	RR	95%CI	*Z*	*P*	Heterogeneity	
						*I*^2^, %	^*^ *P*
Age							
<60	6 [13,35,47,36]	1.29	0.93,1.80	1.52	0.129	79.0	0.000
>60	2 [42,48]	0.99	0.94,1.04	0.34	0.733	0.000	0.603
Race							
Including blacks	4 [42,36,37,48]	0.98	0.94,1.03	0.61	0.542	0.000	0.649
Non-blacks	4 [13,35,47,36]	1.62	1.19,2.22	3.05	0.002	56.7	0.074
Dialysis modality							
PD	2 [35,47]	1.32	0.98,1.79	1.82	0.069	0.000	0.928
HD	6 [13,42,36,37,48]	1.19	0.90,1.55	1.23	0.219	82.1	0.000

*For heterogeneity among subgroups.

RR: relative risk; CI: confidence interval.

### Meta-analysis of the association between circulating TMAO concentrations and cardiovascular mortality

**Figure 3.**
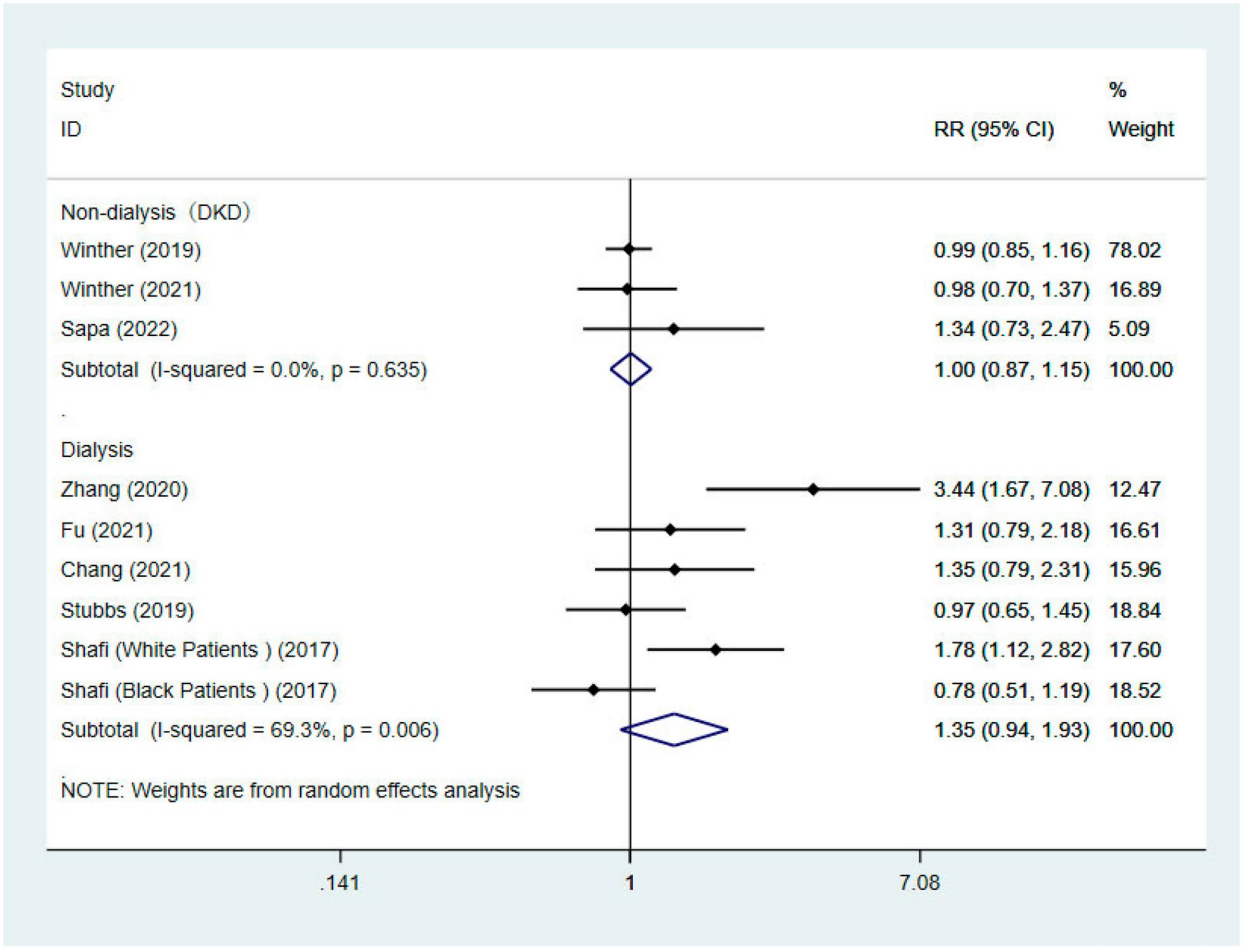
Meta-analysis of the association between circulating TMAO concentrations and cardiovascular mortality in non-dialysis patients and dialysis patients respectively. HR: hazard risk; CI: confidence interval.

9 studies were included to conduct the association of TMAO concentrations and cardiovascular mortality, coincidentally, of which 3 for non-dialysis CKD patients with diabetes complicated by nephropathy [46,39,43] and 6 for dialysis patients [41,42,44,36,37]. A report by Shafi [36] was considered to be 2 independent studies. Consistent with the above results, there was no statistical association between TMAO concentrations and cardiovascular death in patients with diabetes and nephropathy (RR = 1.00, 95%CI = 0.87–1.15, *p* = 0.959, Figure 3). For the remaining 6 studies of dialysis patients, no significant association was also found between TMAO and the risk of cardiovascular mortality (RR = 1.35, 95%CI = 0.94–1.93, *p* = 0.110, Figure 3), and the same results were seen in the subgroup analysis by dialysis modality (Table 4). However, similar to the results of all-cause mortality risk, the result of subgrouping based on race showed that there was a remarkable association between TMAO and cardiovascular mortality in non-black patients (RR = 1.72, 95%CI = 1.19–2.47, *p* = 0.004, Table 4). Of course, heterogeneity was also significantly decreased. Because all studies included patients aged <60, we did not perform subgroup analysis by age (Table 4).

**Table 4.** Subgroup analysis of the association between circulating TMAO concentrations and cardiovascular mortality in dialysis patients.

Subgroup	Studies	RR	95%CI	*Z*	*P*	Heterogeneity	
						*I*^2^, %	^*^ *P*
Race							
Including blacks	2 [36,37]	0.87	0.65,1.17	0.91	0.362	0.000	0.463
Non-blacks	4 [13,35,47,36]	1.72	1.19,2.47	2.89	0.004	44.4	0.145
Dialysis modality							
PD	2 [34,47]	1.33	0.92,1.92	1.51	0.132	0.000	0.937
HD	4 [13,36,37]	1.39	0.80,2.43	1.16	0.248	81.3	0.001

*For heterogeneity among subgroups.

RR: relative risk; CI: confidence inter.

### Dose-response meta-analysis of the association between circulating TMAO concentrations and all-cause mortality

**Figure 4.**
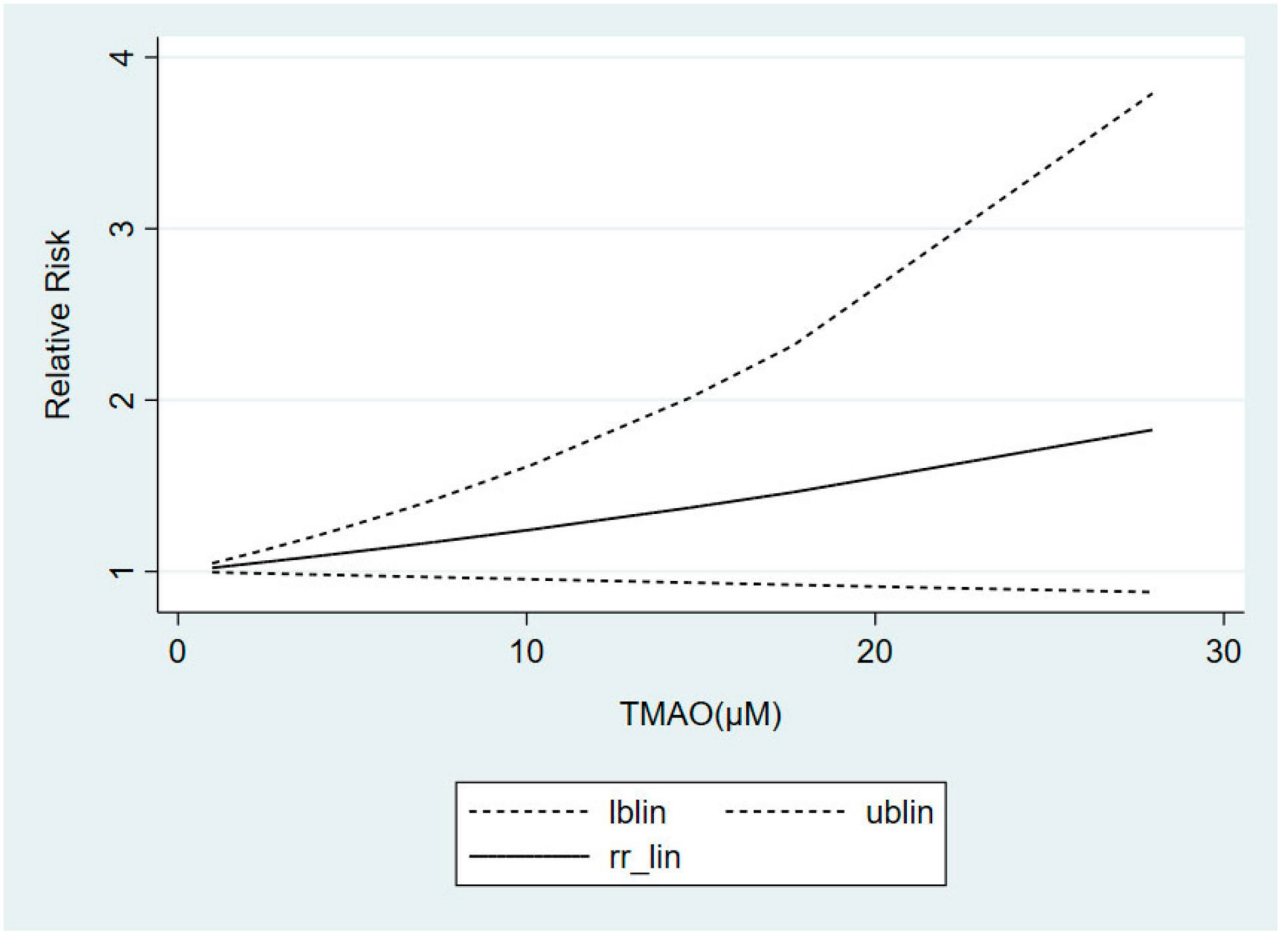
Dose-response meta-analysis of the association between TMAO and all-cause mortality in non-dialysis patients.

**Figure 5.**
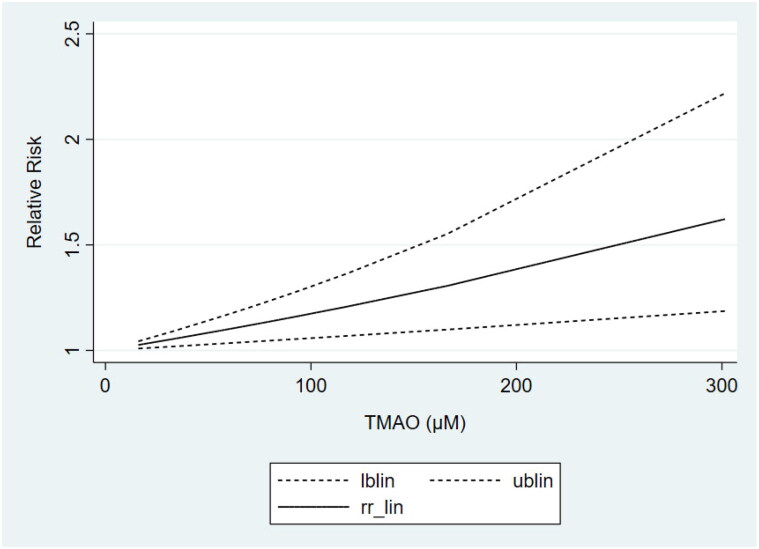
Dose-response meta-analysis of the association between TMAO and all-cause mortality in non-black dialysis patients.

Based on the results of subgrouping, we performed dose-response analyses of studies with ≥3 categories of TMAO concentrations, including 3 articles [24,30,35] on non-dialysis CKD patients and 3 studies [42,44,36] on non-black dialysis patients. Dose-response analyses showed that the TMAO concentrations were linearly related to the risk of all-cause mortality in both non-dialysis CKD (Figure 4) and non-black dialysis patients (Figure 5), which revealed that the risk of all-cause mortality increased gradually with the increase of TMAO concentrations.

### Dose-response meta-analysis of the association between circulating TMAO concentrations and cardiovascular mortality

**Figure 6.**
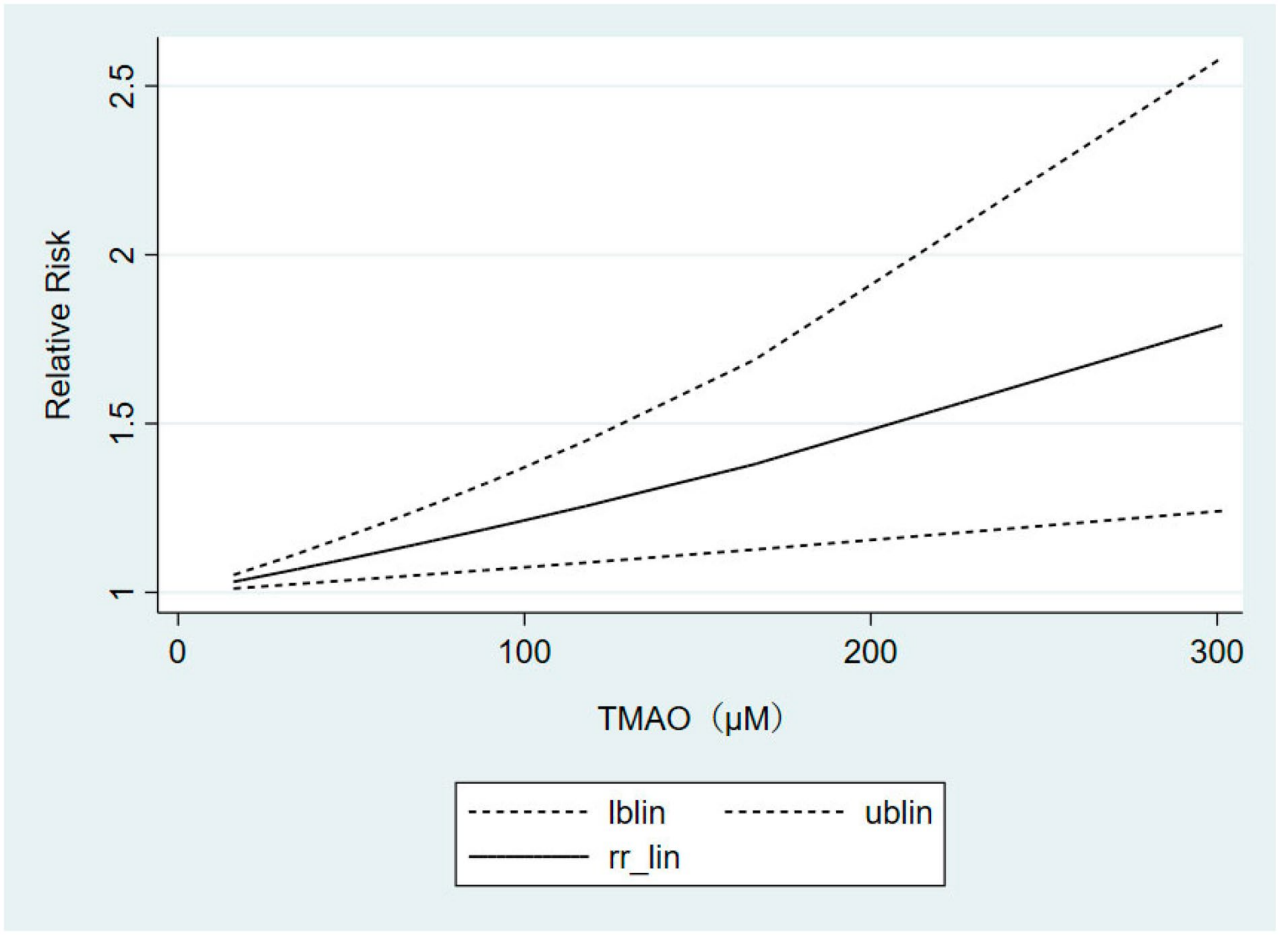
Dose-response meta-analysis of the association between TMAO and cardiovascular mortality in non-black dialysis patients.

The results of the above subgrouping unraveled that TMAO concentrations were strongly correlated with cardiovascular mortality only in the non-black dialysis population. Therefore, we included 3 studies [42,44,36] containing at least 3 categories of TMAO concentrations to perform the dose-response analysis. It also showed a linear relationship between TMAO concentrations and the risk of cardiovascular mortality (Figure 6). That is, higher circulating TMAO concentrations were associated with a greater risk of cardiovascular death in non-black dialysis patients.

### Meta-analysis of the correlations between circulating TMAO concentrations and GFR

**Figure 7.**
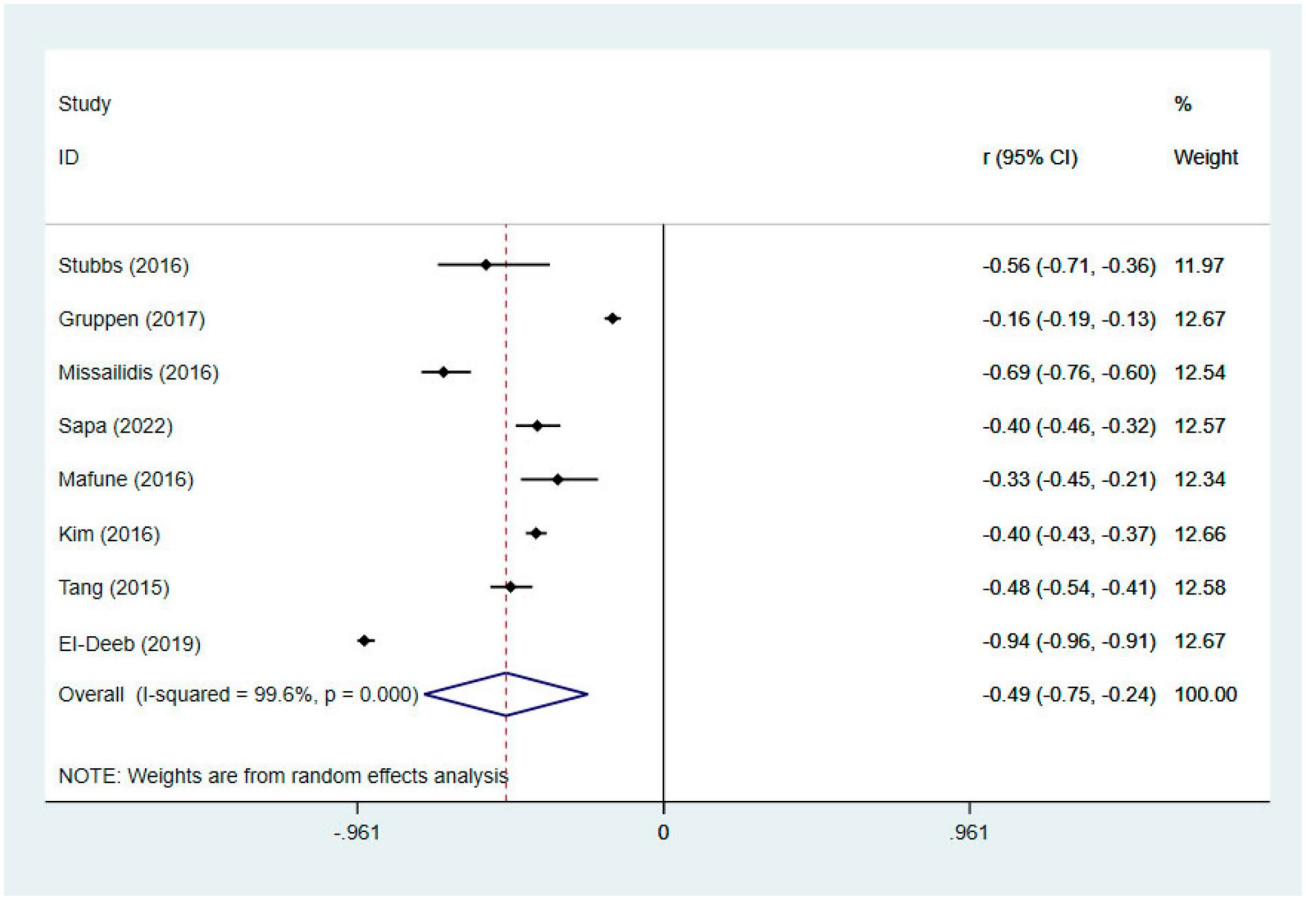
Meta-analysis of the correlations between circulating TMAO concentrations and GFR in non-dialysis CKD patients. *r*, coefficient of association; CI, confidence interval.

We included 8 studies [30,32,22,33,34,35,46,40] involving 9524 individuals to perform the meta-analyses of correlations between TMAO and GFR. Results suggested that circulating TMAO concentrations are strongly correlated with GFR in non-dialysis CKD patients (*r*= −0.49; 95% CI= −0.75, –0.24; *p* <0.001, Figure 7). To examine sources of heterogeneity, and to verify effect sizes across groups, subgrouping was performed according to age (>60 or <60) and GFR levels (>60 or <60). The negative correlation between TMAO concentrations and GFR was stronger among patients with GFR <60 than among those with GFR >60 [GFR <60 (*r*= −0.58; 95% CI= −0.83, –0.33; *p* < 0.001), GFR >60 (*r*= −0.24; 95% CI= −0.40, –0.07; *p* = 0.006), Table 5].

**Table 5.** Subgroup analysis of the correlations between circulating TMAO concentrations and GFR in non-dialysis CKD patients.

Subgroup	Studies	*r*	95%CI	*Z*	*P*	Heterogeneity	
						I^2^, %	^*^ *P*
Age							
<60	4 [26,44,43,49]	–0.59	−1.08, −0.09	2.31	0.021	99.8	0.000
>60	4 [33,41,46,39]	–0.41	–0.46, −0.35	17.11	0.000	53.1	0.094
GFR							
<60	6 [26,41,44,46,39,49]	–0.58	–0.83,–0.33	4.5	0.000	99.3	0.000
>60	2 [33,43]	–0.24	–0.40, −0.07	2.76	0.006	99.6	0.000

*For heterogeneity among subgroups. RR: relative risk; CI: confidence interval.

### Meta-analysis of the correlations between circulating TMAO concentrations and inflammation biomarkers

**Figure 8.**
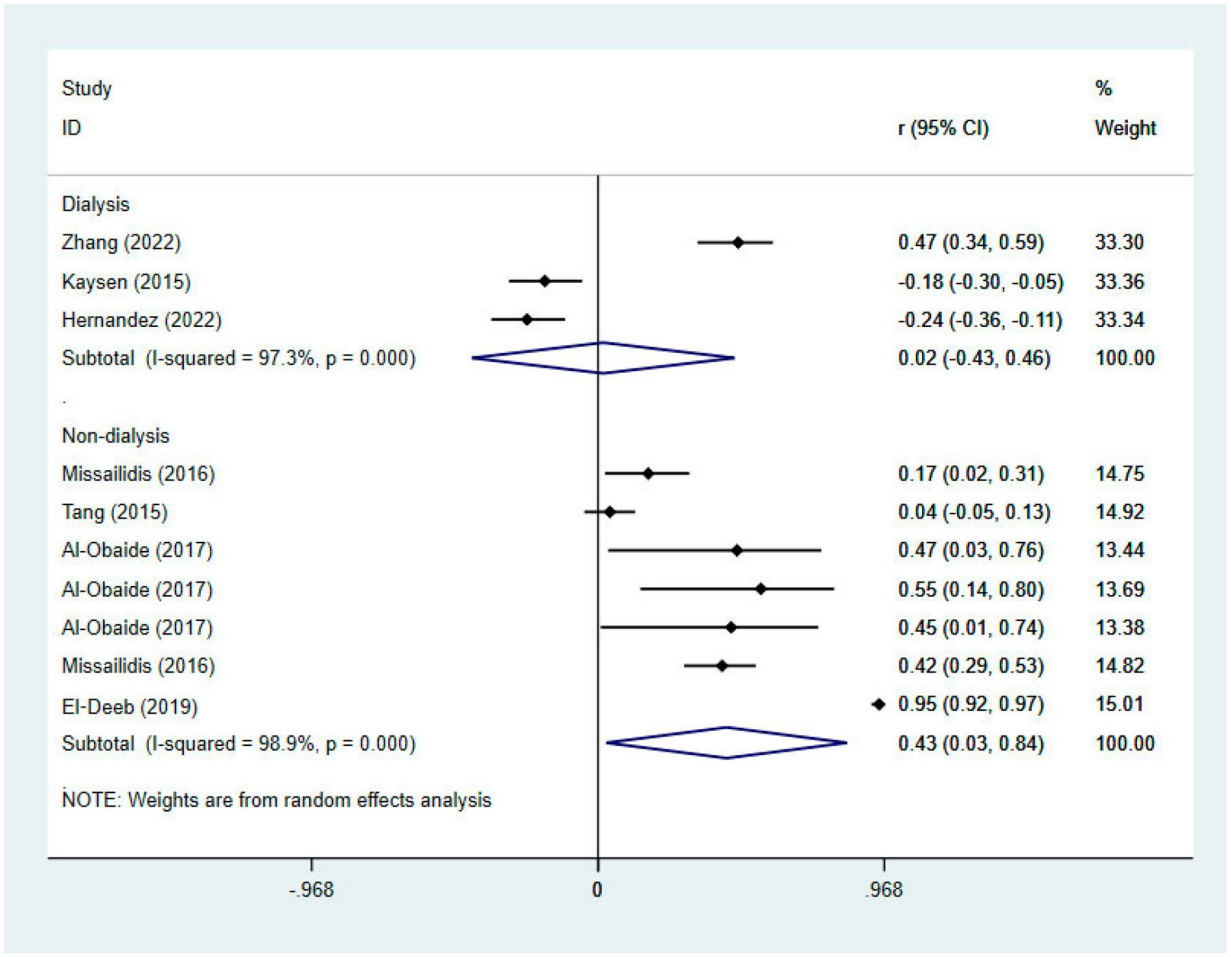
Meta-analysis of the correlations between circulating TMAO concentrations and inflammatory biomarkers in non-dialysis patients and dialysis patients respectively. *r*, coefficient of association; CI, confidence interval.

7 correlation coefficients in 4 studies [30,22,13,40] for non-dialysis patients and 3 correlation coefficients in 3 studies [31,47,45] for dialysis patients were included to perform meta-analyses of the correlations between circulating TMAO concentrations and inflammation biomarkers (Figure 7). A remarkable correlation between TMAO concentrations and inflammation biomarkers was found in non-dialysis patients (*r* = 0.43; 95% CI= 0.03, 0.84; *p* = 0.036, Figure 8). However, in dialysis patients, TMAO concentrations did not appear to be statistically correlated with inflammatory markers (*r* = 0.02; 95% CI= −0.43, 0.46; *p* = 0.937, Figure 8). It’s worth noting that, due to the relatively few studies included for dialysis patients, the results may be greatly biased. Interestingly, we observed that 2 studies [31,45] reported negative correlations between TMAO and inflammatory markers, conversely, 1 reported a positive correlation [47]. These results remind us that more extensive researches are needed to verify the relationship between TMAO and inflammatory markers in dialysis patients and contribute to the clinical treatment scheme.

### Study quality assessment

NOS was used to assess the quality of the included cohort studies with NOS scores of 5-9 (Supplementary Table 2). The AHRQ checklist was used for cross-sectional studies, and the scores were 6–8 (Supplementary Table 3). Almost all studies were judged as moderate or high quality. The QUIPS checklist was used for prognosis studies, and 6 studies were rated as high quality, 10 as moderate quality, and 1 as low quality (Supplementary Table 4).

### Publication bias

The meta-analysis included less than 10 studies for each outcome indicator. Therefore, only a funnel plot was used to detect publication bias (Supplementary Figure 1).

## Discussion

The association between TMAO and mortality has been reported in several previous studies, most recently four months ago [48,49,50]. However, based on including more literature, we have reached further and more precise conclusions in CKD patients. Because of the remarkable difference between dialysis and non-dialysis patients in TMAO concentrations and health status, this is the first report to study the dialysis and non-dialysis CKD patients separately and the first dose-response analyses of the association between circulating TMAO and mortality in CKD patients. Meanwhile, this is also the first systematic review and meta-analysis to focus on ethnic differences in the association of TMAO concentrations and mortality in CKD patients. In addition, we deeply studied the correlation between TMAO and GFR or inflammatory biomarkers to explore the reasons why TMAO caused kidney damage and increased the risk of death. Ultimately, we hope that more precise treatment strategies of dietary modification or drug therapy can be implemented in patients with CKD who are at increased risk of adverse outcomes.

In this study, it was confirmed that elevated TMAO concentrations were associated with increased risk of death in non-dialysis and non-black dialysis CKD patients through both two-class and dose-response analyses, which could be explained by the following mechanisms. First of all, renal fibrosis is the common final outcome of almost all CKD. Administration of choline or TMAO can induce and aggravate renal impairment and renal fibrosis in experimental animals [30,51], while inhibiting the production of TMAO can improve renal pathological injury, protect renal function, and delay the progression of disease in CKD mice [52], suggesting that TMAO is not only a biomarker of impaired renal function but also a potential pathogenic factor that destroys renal function in CKD. Our results of meta-analyses validated this, and found that circulating TMAO concentrations indeed had a clear negative correlation with GFR. The specific mechanism may involve the activation of the TGF-β RI/Smad2 pathway by TMAO, which upregulates the expression of α-SMA and collagen I [53]. TMAO stimulation can induce the proliferation of renal fibroblasts and subsequently increase total collagen expression in a dose-dependent manner with TMAO concentration [54]. Taken together, TMAO can damage renal function by promoting renal fibrosis, therefore, there is a strong correlation between TMAO and GFR, which is consistent with our conclusion of the correlation meta-analysis. However, we found that the association between TMAO and all-cause mortality in almost all studies of non-dialysis patients was adjusted for GFR. The results suggest that the predictive value of circulating TMAO concentrations for the risk of all-cause death is not entirely or directly dependent on the decline in renal function, and may be related to the deterioration of other health conditions caused by the decline in renal function.

As we all know, inflammation is a crucial factor in the progression of CKD, and TMAO has been shown to play a pro-inflammatory role in cardiovascular disease, atherosclerosis, and neurological disorders [55,56,57]. In addition, peritoneal dialysis patients with higher TMAO concentrations have been validated to have a higher risk of developing new-onset peritonitis [47]. Therefore, we performed a meta-analysis of the correlation between TMAO and inflammatory markers in CKD patients. In non-dialysis patients, TMAO concentrations did show strong correlations with inflammatory markers. It may involve the following mechanisms. The elevation of circulating TMAO triggers M1 polarization of macrophages and stimulates the expression of NLRP3 and Caspase-1 to activate the inflammasome [54,58,59]. Meanwhile, TMAO can also activate NFκB and promote the adhesion of leukocytes to the endothelial cells, thereby leading to vascular inflammation [20]. The activation of p38MAPK and the up-regulation of human antigen R are also one of the mechanisms by which TMAO upregulates inflammatory factors, increases the infiltration of inflammatory cells, and causes renal failure [60]. Therefore, inflammation, which is strongly correlated with TMAO in non-dialysis patients, is one of the factors by which high TMAO concentrations increase the risk of death. Apart from these, TMAO can directly contribute to heightened platelet reactivity and increase the thrombosis (heart attack and stroke) risk [38]. TMAO has been found to impair pyruvate and fatty acid oxidation in cardiac mitochondria, leading to disturbances in energy metabolism [61], which may elucidate higher cardiovascular mortality associated with TMAO. Surprisingly, of the only 3 reports on the correlation of TMAO with inflammatory markers, 2 reported a negative correlation. More studies on dialysis patients should be done to minimize bias in trials and reflect the facts.

Our meta-analysis manifested an interesting finding that TMAO concentrations were linearly associated with both all-cause and cardiovascular mortality in non-black dialysis patients, but not in studies involving black patients, which has not been reported in previous meta-analyses. We consulted the relevant literature and wondered whether this result might be caused by differences in TMAO concentrations between different races, and found contradictory results. No statistically significant difference was reported in serum TMAO concentrations between black and white dialysis patients by Shafi [36], whereas the opposite result has been presented in a study on patients with the risk for atherosclerotic cardiovascular disease. It shows that compared with whites, non-white participants were 2.92 times more likely to be in the top quartile of TMAO. However, there are few studies on the difference of blood TMAO concentrations in Asians. In a word, the current evidence on the variability of TMAO concentrations among ethnic can’t explain the results of our meta-analyses, and further research is needed.

In addition to the difference in TMAO concentrations among races, the study by Elaine Ku [62] has shown that black participants are younger and in better health status when dialysis started compared with whites. Thus, the baseline differences lead to a survival advantage for black adults who received dialysis over whites. Beyond those, research on Asians is still lacking. This suggests that in future studies on dialysis mortality in different ethnic groups, we should adjust as much as possible for the effects of underlying diseases and health status.

In brief, TMAO is linearly associated with all-cause mortality in non-dialysis CKD patients and non-black dialysis patients, and also linearly associated with cardiovascular mortality in non-black dialysis patients. Therefore, attention should be paid to controlling the circulating TMAO concentrations of target patients in clinical practice. Because dietary intake is the main source of circulating TMAO, dietary adjustments are considered important. Protein intake at twice the recommended dietary allowance increases the circulating TMAO in healthy older males [63], while a low protein diet declines it in non-dialysis patients [64]. Supplement of oats β-glucan has been demonstrated to be safe and effective in reducing TMAO levels with well tolerated in patients with CKD stages 3-4 [65] [ In addition, elevated plasma TMAO levels are detected in CKD rats transplanted with microbiota obtained from CKD patients [66], so dysbiosis of the intestinal microbiome also plays a key role in the production of TMAO.

Animal experiments confirmed that treatment with butyrate (a short-chain fatty acid originated from dietary fiber) [66], ranitidine, finasteride [67] and rhubarb enema [68] can significantly increase the richness and diversity of some symbiotic bacteria and probiotics, while reduce the abundance of some potential pathogens, thus inhibit the synthesis and release of TMAO, eventually delaying the progression of CKD. However, clinical trials indicate that short-term probiotic supplementation for 3 months has no effect on TMAO levels in HD patients [69]. Long-term clinical observation should be performed to obtain more reliable conclusions. In general, reducing circulating TMAO concentrations by modifying diet and modulating gut microbiota may be a safe and effective therapeutic target to delay CKD progression.

In conclusion, we found a significant positive dose-dependent association between circulating TMAO concentrations and increased all-cause mortality in non-dialysis and non-black dialysis CKD patients. Likewise, TMAO levels are positively correlated with the cardiovascular mortality risk in a dose-dependent manner in patients with non-black dialysis. However, more prospective studies are still needed to elucidate the relationship between TMAO concentration and mortality in CKD patients with different dialysis status and different races, to help make better clinical decisions.

## Limitations

The current study has several limitations. First, because dialysis and non-dialysis patients were studied separately, the meta-analysis for each effect size included less than 10 articles, which may result in large errors in the publication bias. In addition, the loss of some data could increase publication bias. Second, the heterogeneity of studies in some subgroups remains high, and we cannot explain the full sources of heterogeneity. Third, there is too little data on the relationship between TMAO concentrations and the risk of cardiovascular mortality in non-dialysis patients, and the underlying diseases are relatively single, hence more researches are needed for a more reliable meta-analysis. Fourth, in dose-response meta-analyses, the death risk associated with the highest level of exposure is typically analyzed [70]. However, as the highest exposure levels vary among studies, prospective studies with larger samples are needed to demonstrate values of TMAO concentrations that significantly increase the risk of death in CKD patients. Fifth, there are too few studies in the literature on the correlation between TMAO concentration and inflammation in dialysis patients to draw objective results, and because of this, subgroup analysis was not performed. In a word, these results should be interpreted with caution and treated as hypotheses generating.

## Supplementary Material

Supplemental Material

## Data Availability

The raw data supporting the conclusions of this article will be made available by the authors, without undue reservation.
