# Supplementary material for: Gut microbiota-derived trimethylamine N-oxide is associated with the risk of all-cause and cardiovascular mortality in patients with chronic kidney disease: a systematic review and dose-response meta-analysis
Source: Ann Med. 2023 May 29;55(1):2215542. doi: 10.1080/07853890.2023.2215542 (PMC10228303; doi:10.1080/07853890.2023.2215542)
Supplement: Supplemental Material [file IANN_A_2215542_SM9605.pdf]

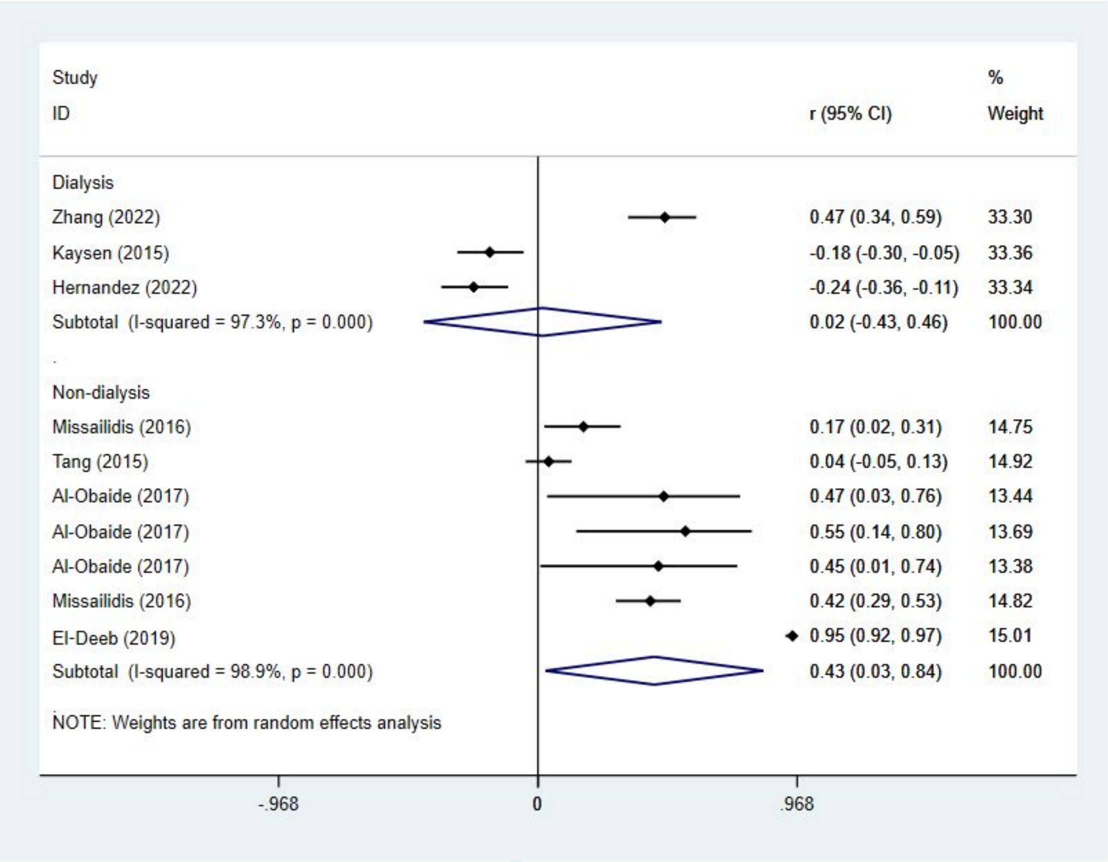

Figure 8. Meta-analysis of the correlations between circulating TMAO concentrations and inflammatory biomarkers in non-dialysis patients and dialysis patients respectively. *r*, coefficient of association; CI, confidence interval.

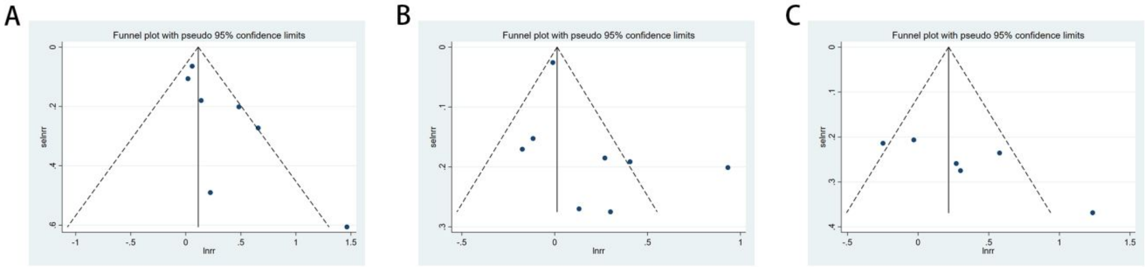

Figure S1 Begg funnel plots (with pseudo 95% CI) for studies evaluating

the association between TMAO and (A)all-cause mortality in non-dialysis CKD patients, (B)all-cause mortality in dialysis patients, and (C)cardiovascular mortality in dialysis patients.

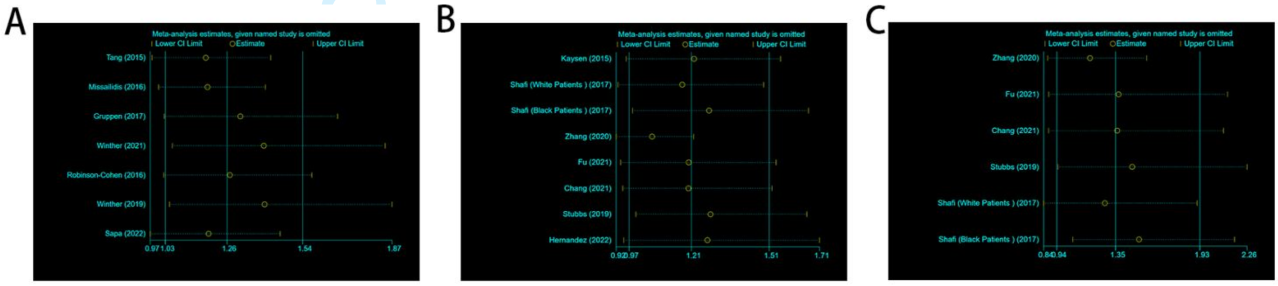

Figure S2 Sensitivity analyses for studies evaluating the association between TMAO and (A)all-cause mortality in non-dialysis CKD patients, (B)all-cause mortality in dialysis patients, and (C)cardiovascular mortality in dialysis patients.

Supplementary Table 1 Search strategy

	**Search terms**
PubMed	#1 trimethylamine oxide OR TMAO [MeSH Terms]<br>#2 trimethylamine oxide OR TMAO [Title/Abstract]<br>#3 #1 OR #2<br>#4 kidney disease OR CKD OR ESKD OR ESRD OR dialysis [Title/Abstract]<br>#5 #3 AND #4
EMBASE	Title-Abstract-Author Keywords (('trimethylamine oxide' OR 'TMAO') AND ('kidney disease' OR 'CKD' OR 'ESKD' OR 'ESRD' OR 'dialysis'))
Web of Science	TS= (('trimethylamine oxide' OR TMAO) AND ('kidney disease' OR CKD OR ESKD OR ESRD OR dialysis))

For Review Only

For Peer Review Only

Supplementary Table 2 Newcastle–Ottawa quality assessment scale (NOS) for cohort study

Authors	Year	Journal	Selection				Comparability of cohorts on the basis of the design or analysis	Outcome			Final score
			Representativeness of the exposed cohort	Selection of the non-exposed cohort	Ascertainment of exposure	Demonstration that outcome of interest was not present at start of study		Assessment of outcome	Was follow-up long enough for outcomes to occur	Adequacy of follow up of cohorts	
Tang et al.	2015	*N Engl J Med*	-	*	*	*	-	*	*	-	5
Kaysen et al.	2015	*J Am Heart Assoc*	*	*	*	*	-	*	-	*	6
Stubbs et al.	2016	*J Am Soc Nephrol*	-	*	*	*	**	*	*	-	7
Missailidis et al.	2016	*PLoS One*	*	*	*	*	-	*	*	-	6
Robinson-Cohn et al.	2016	*PLoS One*	*	*	*	*	-	*	-	-	5
Kim et al.	2016	*Kidney Int*	*	*	*	*	-	*	-	-	5
Gruppen et al.	2017	*Sci Rep*	*	*	*	*	**	*	*	-	8
Shafi et al.	2017	*J Am Soc Nephrol*	*	*	*	*	-	*	-	*	6
Stubbs et al.	2019	*Clin J Am Soc Nephrol*	*	*	*	*	**	*	*	-	8
Winther et al.	2019	*Diabetes Care*	*	*	*	*	**	*	*	*	9
Zhang et al.	2020	*Ren Fail*	*	*	*	*	**	*	*	*	9
Fu et al.	2021	*Blood Purif*	*	*	*	*	**	*	*	*	9
Winther et al.	2021	*PLoS One*	*	*	*	*	**	*	*	*	9
Chang et al.	2021	*Perit Dial Int*	*	*	*	*	**	*	-	*	8
Sapa et al.	2022	*Am J Kidney Dis*	*	*	*	*	**	*	*	-	8
Hernandez et al.	2022	*Sci Rep*	*	*	*	*	-	*	*	-	6
Zhang et al.	2022	*Transl Res*	*	*	*	*	**	*	-	*	8

Supplementary Table 3 Agency for Healthcare Research and Quality (AHRQ) for cross-sectional study

	Mafune et al. 2016	A. I. Al-Obaide et al. 2017	EI-Deeb et al. 2019
1) Define the source of information (survey, record review)	Y	Y	Y
2) List inclusion and exclusion criteria for exposed and unexposed subjects (cases and controls) or refer to previous publications	Y	Y	Y
3) Indicate time period used for identifying patients	Y	Y	Y
4)Indicate whether or not subjects were consecutive if not population-based	Y	Y	Y
5) Indicate if evaluators of subjective components of study were masked to other aspects of the status of the participants	U	U	U
6) Describe any assessments undertaken for quality assurance purposes (e.g., test/retest of primary outcome measurements)	Y	Y	Y
7) Explain any patient exclusions from analysis	Y	N	N
8) Describe how confounding was assessed and/or controlled.	Y	Y	Y
9) If applicable, explain how missing data were handled in the analysis	U	U	U
10) Summarize patient response rates and completeness of data collection	Y	U	U
11) Clarify what follow-up, if any, was expected and the percentage of patients for which incomplete data or follow-up was obtained	U	U	U
Final score	8	6	6

Supplementary Table 4 the Quality In Prognosis Studies (QUIPS) tool for prognosis studies

**Study**			**Study Participation**	**Study Attrition**	**Prognostic Factor Measurement**	**Outcome Measurement**	**Study Confounding**	**Statistical Analysis and Reporting**	**Final quality rating**
Tang et al.	2015	*N Engl J Med*	moderate	moderate	moderate	low	moderate	low	moderate
Kaysen et al.	2015	*J Am Heart Assoc*	low	moderate	low	low	high	low	low
Missailidis et al.	2016	*PLoS One*	low	low	low	low	low	low	high

For Peer Review Only

Kim et al.	2016	*Kidney Int*	low	moderate	low	low	moderate	low	moderate
Robinson-Cohn et al.	2016	*PLoS One*	low	moderate	low	low	moderate	moderate	moderate
Stubbs et al.	2016	*J Am Soc Nephrol*	moderate	moderate	moderate	low	moderate	low	moderate
Gruppen et al.	2017	*Sci Rep*	low	low	low	low	low	low	high
Shafi et al.	2017	*J Am Soc Nephrol*	low	moderate	moderate	low	moderate	low	moderate
Winther et al.	2019	*Diabetes Care*	low	low	low	low	low	low	high
Stubbs et al.	2019	*Clin J Am Soc Nephrol*	low	moderate	low	low	moderate	low	moderate
Zhang et al.	2020	*Ren Fail*	low	low	low	low	low	low	high
Fu et al.	2021	*Blood Purif*	low	low	low	low	moderate	low	moderate
Winther et al.	2021	*PLoS One*	low	moderate	low	low	moderate	low	moderate
Chang et al.	2021	*Perit Dial Int*	low	low	low	low	low	low	high
Sapa et al.	2022	*Am J Kidney Dis*	low	low	low	low	low	low	high
Hernandez et al.	2022	*Sci Rep*	low	moderate	low	low	moderate	low	moderate
Zhang et al.	2022	*Transl Res*	low	moderate	low	low	moderate	low	moderate

High quality: risk of bias was rated low on at least four of the six domains and was rated low for both study attrition and study confounding (shaded).

Moderate quality: risk of bias was rated low or moderate on at least four of the six domains and was rated moderate for either study attrition or study confounding (shaded).

Low quality: risk of bias was rated high on at least four of the six domains and/or was related high for study attrition and study confounding (shaded).

Studies with high risk of bias for study attrition or study confounding were rated as low quality.

For Peer Review Only
